# Association between Dopamine Receptor D2 (DRD2) Variations rs6277 and rs1800497 and Cognitive Performance According to Risk Type for Psychosis: A Nested Case Control Study in a Finnish Population Sample

**DOI:** 10.1371/journal.pone.0127602

**Published:** 2015-06-26

**Authors:** Hugh Ramsay, Jennifer H. Barnett, Jouko Miettunen, Sari Mukkala, Pirjo Mäki, Johanna Liuhanen, Graham K. Murray, Marjo-Riitta Jarvelin, Hanna Ollila, Tiina Paunio, Juha Veijola

**Affiliations:** 1 Department of Psychiatry, Centre for Clinical Neuroscience, University of Oulu, Oulu, Finland; 2 Health Service Executive, Dublin, Ireland; 3 Department of Psychiatry, University of Cambridge, Cambridge Biomedical Campus, Cambridge, United Kingdom; 4 Cambridge Cognition Ltd., Bottisham, Cambridge, United Kingdom; 5 Institute of Health Sciences, University of Oulu, Oulu, Finland; 6 Medical Research Center Oulu, University of Oulu and Oulu University Hospital, Oulu, Finland; 7 Department of Psychiatry, Oulu University Hospital, Oulu, Finland; 8 Department of Psychiatry, Länsi-Pohja healthcare district, Finland; 9 Department of Psychiatry, the Middle Ostrobothnia Central Hospital, Kiuru, Finland; 10 Mental health services, Joint Municipal Authority of Wellbeing in Raahe District, Finland; 11 Mental health services, Basic Health Care District of Kallio, Finland; 12 Visala Hospital, the Northern Ostrobothnia Hospital District, Finland; 13 Public Health Genomics Unit, National Institute for Health and Welfare and Institute for Molecular Medicine, Helsinki, Finland; 14 Department of Public Health Science and General Practice, Institute of Health Sciences, University of Oulu, Oulu, Finland; 15 Faculty of Medicine, School of Public Health, Imperial College London, London, United Kingdom; 16 Department of Psychiatry, Institute of Clinical Medicine, University of Helsinki and Helsinki University Central Hospital, Helsinki, Finland; Mayo Clinic, UNITED STATES

## Abstract

**Background:**

There is limited research regarding the association between genes and cognitive intermediate phenotypes in those at risk for psychotic disorders.

**Methods:**

We measured the association between established psychosis risk variants in dopamine D2 receptor (DRD2) and cognitive performance in individuals at age 23 years and explored if associations between cognition and these variants differed according to the presence of familial or clinical risk for psychosis. The subjects of the Oulu Brain and Mind Study were drawn from the general population-based Northern Finland 1986 Birth Cohort (NFBC 1986). Using linear regression, we compared the associations between cognitive performance and two candidate DRD2 polymorphisms (rs6277 and rs1800497) between subjects having familial (n=61) and clinical (n=45) risk for psychosis and a random sample of participating NFBC 1986 controls (n=74). Cognitive performance was evaluated using a comprehensive battery of tests at follow-up.

**Results:**

Principal components factor analysis supported a three-factor model for cognitive measures. The minor allele of rs6277 was associated with poorer performance on a verbal factor (p=0.003) but this did not significantly interact with familial or clinical risk for psychosis. The minor allele of rs1800497 was associated with poorer performance on a psychomotor factor (p=0.038), though only in those at familial risk for psychotic disorders (interaction p=0.049).

**Conclusion:**

The effect of two DRD2 SNPs on cognitive performance may differ according to risk type for psychosis, suggesting that cognitive intermediate phenotypes differ according to the type (familial or clinical) risk for psychosis.

## Introduction

Psychotic disorders, especially schizophrenia, have increasingly been recognised as complex neurodevelopmental disorders that are commonly preceded by a significant prodromal period mainly in adolescence and young adulthood [1]. While much about the pathophysiology of psychotic disorders remains unknown, genetic research has examined endophenotypes or intermediate phenotypes. Intermediate phenotypes are associated with the illness, heritable, primarily state independent and co-segregate within families [2]. Further knowledge of how intermediate phenotypes evolve has the potential to inform prevention strategies.

Both psychotic disorders and their prodrome have been associated with poorer cognitive performance [3]. Furthermore, poorer cognitive performance has been associated with adverse long-term functional [4,5] and symptomatic [6] outcomes in those with psychotic disorders. Cognitive impairments have been demonstrated across a wide range of domains in schizophrenia, including attention, executive function, spatial ability, verbal learning and memory [7]. Similar deficits have been shown in those at high risk for psychosis, including attention, verbal learning, executive function, processing speed and verbal and nonverbal working memory [8–14]. The Consortium on the Genetics of Schizophrenia evaluated several candidate neurocognitive intermediate phenotypes for schizophrenia and selected attention, verbal memory and working memory as implicated in the pathophysiology of schizophrenia [15]. Indeed, there have been numerous findings in relation to cognitive measures as intermediate phenotypes for schizophrenia [16].

Dopamine neuron projections to the prefrontal cortex in the mesocortical dopamine system play an essential role in several aspects of normal cognitive function [17]. Indeed decreased dopaminergic neurotransmission appears to contribute to cognitive deficits in schizophrenia, particularly in the areas of executive functions and working memory [18]. However, precise regulation of prefrontal dopaminergic tone is essential as excess dopamine may also be harmful to cognition. The dopamine D2 receptor is a G-protein coupled receptor inhibiting adenylyl cyclase activity involved in mesocorticolimbic pathways [19]. The Dopamine D2 receptor (DRD2) is a target for antipsychotic drugs in the treatment of schizophrenia [20] and genetic variation within and adjacent to DRD2 has been associated with schizophrenia [21,22], including in a recent large genome-wide association study [23]. This receptor has also been associated with abnormalities in functioning in the prefrontal cortex [24] and higher rates of D2 receptor occupancy by antipsychotic drugs has been associated with poorer cognitive performance, particularly in vigilance [25]. The DRD2 gene has also been associated with negative symptoms and poorer performance on a sustained attention task [26].

There have been a variety of studies examining cognitive associations at the DRD2 SNPs, rs6277 and rs1800497. In the case of the rs6277 polymorphism, the T allele has been associated with better learning from positive and negative outcomes in a young adult sample [27], while the CC genotype has been associated with poorer overall cognitive ability in an older Scottish population sample [28], possibly suggesting the learning those with T alleles may better learn into older age. Biologically, T alleles at rs6277 have been associated with decreased affinity of D2 receptors in the striatum [29]. The rs1800497 polymorphism has also been associated with cognitive abilities. Those with the minor (T) allele perform more poorly in memory tasks [30–32] and other cognitive tasks [33,34] compared to those without a T allele. Interestingly, in an older Scottish population sample, those who are heterozygous showed the poorest overall cognitive ability [28]. At a receptor level, the minor T allele is associated with reduced dopamine binding sites in the brain [35]. These associations between SNPs at DRD2 and cognition may be even more significant in those at risk for psychosis, though research in this area is lacking.

While DRD2 SNPs have been associated with cognitive intermediate phenotypes in general population samples and with schizophrenia itself, their association with cognitive outcomes in samples at high risk for psychotic disorders and whether these differ from the general population have not been examined. One imaging study suggested differences in the correlation between dopamine receptor density and activation in the right middle frontal gyrus between those at clinical risk for psychosis and controls [36], but there has been no research on differences according to DRD2 SNPs. We hypothesise that those at increased risk for psychosis should show stronger associations between DRD2 SNP variants associated with cognitive difficulties in other groups (C allele at rs6277 and T allele at rs1800497) and adverse cognitive outcomes than those not at risk for psychosis.

## Materials and Methods

### Participants

The sample consists of participants in the Northern Finland 1986 Birth Cohort (NFBC 1986). This cohort included 99% of those born between July 1985 and June 1986 (9,432 live-born children) in the two northernmost regions of Finland (Oulu and Lapland) [37]. This cohort was surveyed prospectively during gestation and it has continued since. The Ethics committee of the Northern Ostrobothnia Hospital District in Finland has approved the study.

A subsample, the Oulu Brain and Mind study, of the NFBC 1986 members was invited to participate in a field study during 2007–2010 based on previously collected data. Participants were aged 21–25 years, with a mean age being 23 years. The characteristics of this sample have been described elsewhere [38]. Register data included psychosis diagnosis, which was obtained from the nationwide Finnish Hospital Discharge Register (FHDR) of NFBC 1986 members and their parents, and information regarding the right to reimbursable medication due to psychotic disorders as recorded in the registers of the Social Insurance Institute in the cohort members. Psychosis diagnosis and final classification of participants as at risk for psychosis were confirmed in the Structured Interview for Prodromal Syndromes (SIPS) [39] together with the Structured Clinical Interview for DSM IV axis-I disorders (SCID). The SIPS identified the presence of attenuated psychotic symptoms, brief, limited and intermittent psychotic symptoms and/or genetic risk with recent functional deterioration. These clinical interviews and cognitive tests were performed on a single day of assessment. The final groups for the present study, defined according to SIPS interview, were: familial risk for psychosis group (n = 61) with one parent or both with psychosis, clinical risk (prodromal syndrome) for psychosis group (n = 47) and control group, a random sample of participating NFBC 1986 members (n = 74). Fourteen individuals fulfilled both familial and clinical risk for psychosis and they were classified with the clinical risk group because they were thought to be at higher risk for developing psychosis based on prodromal symptoms of psychosis. The total sample was therefore 182 individuals. Full details of those included has been reported elsewhere [38,40,41]. Subjects with psychotic disorders were not included as group numbers were too low to allow for meaningful comparison of associations. All participants provided written informed consent.

The combined familial risk group, clinical risk group and control group were examined for genetic associations with cognitive outcomes. Among the 74 controls, there was SNP data for analysis for 64 individuals for rs6277 and 64 individuals for rs1800497. Among the 45 participants with clinical risk, there was SNP data for analysis for 38 individuals both SNPs. Among the 61 participants with familial risk, there was SNP data for analysis for 56 individuals. This meant there was a total sample for analysis of 158 individuals.

### Assessments

#### Cognitive assessments

A number of cognitive assessments were performed on participants, assessing verbal and non-verbal intellectual ability, learning and memory, executive functioning, working memory, attention, decision-making and fine motor functioning. Intellectual ability was tested using vocabulary and matrix reasoning sections of the Wechsler Adult Intelligence Scale (WAIS-3) [42]. Learning and memory were assessed using the Logical Memory component (immediate and delayed parts) of the Wechsler Memory Scale-Revised (WMS-R) [43] and the Paired Associates Learning (PAL) test from the CANTAB battery [44]. Executive functioning/working memory was measuring using Digit Span Backwards [42], Semantic Fluency [45] and Stockings of Cambridge (SOC) [46]. Working memory/attention was assessed using Digit Span Forwards [42] and Rapid Visual Information Processing (RVP) [47]. Fine motor functioning was measured with the Grooved Pegboard [48].

Raw scores of neurocognitive ability tests that were normally distributed (except for PAL) were transformed to Z-scores using means and standard deviations derived from the control group. Prior to conversion to Z-scores non-normally distributed variables were transformed. Paired associates learning (PAL) was transformed using a square root transformation. Following this, factor analysis with principal components factoring was performed on variables with complete data available. This method was used under the assumption that the tests were examining latent neurocognitive factors and resulted in the identification of three main factors (presented in Table 1 below).

**Table 1:** Loadings of baseline performance for individual tests to three main factors (n = 181).

Variable	Factor 1: “Verbal performance”	Factor 2: “Psychomotor performance”	Factor 3: “Non-verbal performance”	Uniqueness
Vocabulary	0.61	-0.39	0.28	0.41
Matrix reasoning	0.26	0.02	0.75	0.37
Logical memory	0.32	-0.29	0.57	0.49
Paired Associates Learning	-0.09	0.21	-0.71	0.44
Digit span backwards	0.78	-0.08	0.26	0.31
Verbal fluency	0.46	-0.51	0.24	0.47
Stockings of Cambridge	-0.09	-0.17	0.69	0.49
Digit span forwards	0.88	-0.05	-0.05	0.23
Pegboard dominant	-0.13	0.91	-0.07	0.16
Pegboard non-dominant	-0.02	0.85	-0.11	0.27

#### SNP analysis

Two candidate SNPs from DRD2 were selected based on previous association with schizophrenia and related phenotypes [21,22,28,49,50]. Genotyping was performed at the Institute for Molecular Medicine Finland with Sequenom Mass array technology (Sequenom, San Diego, California). SNPs had success rate >95%, minor allele frequencies of 0.17 for rs1800497 and 0.44 for rs6277 and did not deviate from Hardy-Weinberg equilibrium in the control population (P>0.05). Based on the findings discussed above, the SNP rs6277 was analysed as a continuous variable with those with the TT genotype as the baseline group. In the case of the rs1800497 SNP, those with any T allele (homozygous or heterozygous) were compared with the baseline CC group.

#### Background variables

Information on gender and education level of cohort members was collected. Educational level was based on a question about basic education and categorized into three classes: less than nine school years, more than nine school years without exit examination and completion of school exit examination [38].

### Statistical methods

Stata 11 was used for statistical analyses. We first examined if SNP status was directly associated with risk group (familial risk, clinical risk) compared with controls, education status and gender using chi-squared tests. We also examined whether specific risk group (familial vs. clinical risk) was associated with the three cognitive factors identified in factor analysis. Factor analysis was used in order to reduce the number of tests performed and therefore reduce the risk of false positive results. Factor analysis using principal component factor was used because we did not have a pre-defined idea of the structure or number of variable dimensions [51]. The factors were then varimax rotated to produce orthogonal factors that were not correlated to each other. Prior to performing our main analysis to examine the association between the SNPs of interest and cognitive outcomes, we tested if there was sufficient sample size to detect a medium effect size in our analyses. By convention, an f value of 0.10 for effect size is small, 0.25 is medium and 0.40 is large [52]. Using G*Power software, 158 individuals are needed to provide 80% power to detect a medium effect size (f = 0.25) for a 3X2 interaction (numerator degrees of freedom = 2, number of groups = 6) between risk group and SNP allele load in predicting cognitive performance [53,54]. However, we lacked power to detect particularly small effect sizes (f≤0.10). In these primary analyses, in the case of rs6277 we examined whether the number of C alleles was associated with each quantitative outcome (allele load or allele-based additive models), using linear regression, controlling for gender, education and risk group for psychosis. In the case of rs1800497, we examined whether the presence of any T allele was associated with each quantitative outcome, using linear regression, controlling for gender, education and risk group for psychosis. Where we found a direct association, we further tested for interaction with risk group for psychosis and compared models with and without interaction using the chi-squared test for interaction. Where a SNP was associated with a cognitive factor, the magnitude of this association was measured by determining its Cohen’s f^2^ value in each subgroup to clarify if associations were similar across these groups.

## Results

**Fig 1:**
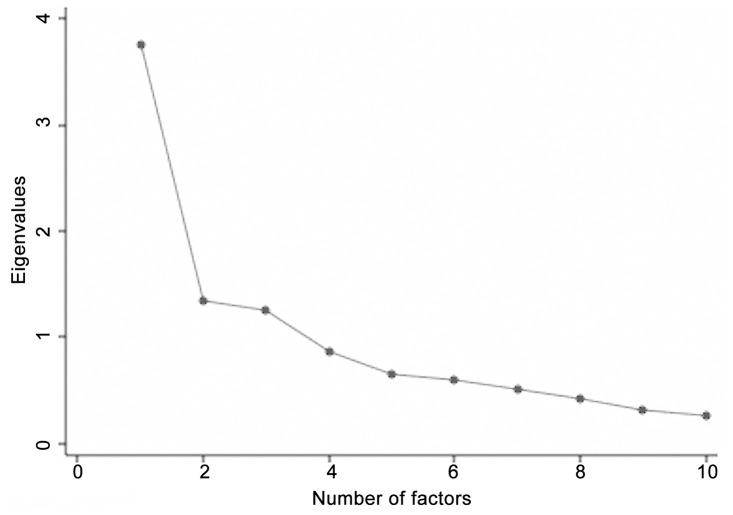
Scree plot of Eigenvalues for principle component factor analysis of neurocognitive variables.

Principal components factor analysis of cognitive performance showed three eigenvalues >1, suggesting a three factor neurocognitive model (presented in Scree plot in Fig 1). These three factors accounted for 64% of performance variance. The loadings of the ten cognitive tests to the three factors are presented in Table 1. Broadly, WAIS vocabulary and verbal working memory (the digit span tasks) loaded best to factor 1 with some contribution from verbal fluency. We therefore termed this factor “verbal performance”. The pegboard tasks loaded best to factor 2, with a weaker loading also from verbal fluency. We termed this factor “psychomotor performance”. Matrix reasoning, PAL and SOC loaded best to factor 3, with weaker loading from logical memory. We termed this factor “non-verbal performance”.

The SNPs were not associated with either risk group for a psychotic disorder or gender or education status (see Table 2).

**Table 2:** Association between SNP minor alleles and risk groups for a psychotic disorder, and gender and education.

Variable		rs6277 C allele^		rs1800497 T allele#	
		OR (95% CI)	P-value	OR (95% CI)	P-value
Risk group (n = 157–158)	Controls	1.00	N/A	1.00	N/A
	Familial risk	0.87 (0.33–2.27)	0.773	0.80 (0.36–1.78)	0.592
	Clinical risk	1.25 (0.39–3.97)	0.710	1.02 (0.43–2.41)	0.972
Gender (n = 157–158)	Male	1.00	N/A	1.00	N/A
	Female	1.84 (0.78–4.34)	0.167	1.37 (0.67–2.79)	0.385
Education (n = 156–157)	Elementary	1.00	N/A	1.00	N/A
	High school	0.54 (0.22–1.34)	0.183	0.80 (0.40–1.60)	0.529

OR = odds ratio; 95% CI = 95% confidence interval; N/A = not applicable.

^^^As continuous variable.

^#^Comparing those with 1–2 T alleles to those without a T allele.

### SNPs and neurocognitive performance

The C allele of rs6277 was associated with better performance in the verbal factor (P = 0.003) (see Table 3) but no difference in performance in the nonverbal factor (P = 0.463) or the psychomotor factor (P = 0.064) in the entire sample. Further analysis for interaction between the minor allele (T) and type of risk for psychosis indicated the absence of significant interaction according to risk group (P = 0.255 on chi-squared test for interaction). Subgroup analyses indicated that the C allele showed a consistent strong association with better performance on the verbal factor among those with familial risk (Cohen’s f^2^ = 2.226, P = 0.011) and among those with clinical risk (Cohen’s f^2^ = 3.989, P = 0.014) but not among the control group (Cohen’s f^2^ = 0.051, P = 0.649). These suggest the possibility of more subtle interactions than those detected by the conservative chi-squared test for interaction (see Table 3). Table 4 illustrates the stratified mean scores on each factor according to risk and genotype group and the number within each group, which do not adjust for gender or education.

**Table 3:** Association between polymorphisms and cognitive outcomes, controlling for gender and education level, in the entire sample of those at risk for psychosis and population controls.

	rs6277			rs1800497		
Cognitive variable	Beta	Effect size (Cohen’s f^2^)	P-value	Beta	Effect size (Cohen’s f^2^)	P-Value
Verbal factor performance	**0.327**	**1.03**	**0.003**	-0.102	N/A	0.539
Psychomotor factor performance	0.208	N/A	0.064	**0.332**	**+0.055**	**0.038**
Non-verbal factor performance	0.083	N/A	0.463	0.138	N/A	0.417

**Table 4:** Associations between the rs6277 minor allele and verbal factor performance and rs1800497 minor allele and psychomotor factor performance, according to risk type for psychosis.

SNP and cognitive factor	Risk group	Beta	Effect size (Cohen’s f^2^)	P-Value
rs6277 and verbal factor performance	Controls	0.081	+0.051	0.649
	Familial risk	**0.478**	**+2.226**	**0.011**
	Clinical risk	**0.592**	**+3.989**	**0.014**
rs1800497 and psychomotor factor performance	Controls	-0.053	-0.121	0.822
	Familial risk	**0.838**	**+0.564**	**0.002**
	Clinical risk	0.351	+0.466	0.401

The presence of one ore more T alleles of rs1800497 was associated with poorer performance in the psychomotor factor (P = 0.038) but no difference in performance in the verbal factor (P = 0.539) or the non-verbal factor (P = 0.417). Further analysis for interaction between the minor allele and type of risk for psychosis indicated significant interaction according to risk group (P = 0.049 on chi-squared testing for interaction). This was confirmed on subgroup analysis (see tables) where having one or more T alleles at rs1800497 was associated with a strongly poorer performance in the psychomotor factor among those with familial risk (Cohen’s f^2^ = 0.564, P = 0.002). The association was similar in magnitude and direction in the clinical risk group but was not statistically significant (Cohen’s f^2^ = 0.466, P = 0.401), while this direction of effect was not evident in the control group (Cohen’s f^2^ = -0.121, P = 0.822). Table 5 indicates these differences in more detail with stratified mean scores on each factor according to SNP genotype and risk group, without adjusting for gender or education.

**Table 5:** Mean performance scores according to SNP genotype, unadjusted for gender or education.

SNP & Risk group	SNP Variant	Group number	Verbal performance	Psychomotor performance	Non-verbal performance
			Mean (SD)	Mean (SD)	Mean (SD)
rs6277 controls	TT	18	0.34 (0.77)	0.04 (0.72)	0.18 (0.80)
	CT	34	-0.20 (1.08)	-0.14 (1.10)	0.07 (0.93)
	CC	10	0.19 (1.08)	0.02 (1.04)	-0.39 (0.87)
rs6277 familial risk	TT	19	0.35 (1.01)	0.33 (1.03)	-0.20 (0.96)
	CT	27	-0.40 (0.91)	-0.09 (1.03)	-0.20 (1.03)
	CC	10	-0.07 (0.96)	-0.08 (0.67)	0.23 (1.01)
rs6277 clinical risk	TT	13	0.55 (0.96)	0.22 (0.76)	0.58 (0.77)
	CT	20	-0.10 (0.92)	0.24 (1.44)	-0.11 (1.08)
	CC	5	-0.62 (0.66)	-0.52 (0.68)	0684 (0.64)
rs1800497 controls	CC	44	0.12 (1.02)	-0.03 (1.02)	0.03 (0.93)
	CT	17	-0.21 (1.03)	-0.13 (0.93)	-0.07 (0.77)
	TT	2	0.03 (0.01)	-0.44 (0.54)	1.02 (0.07)
rs1800497 familial risk	CC	41	-0.05 (1.08)	-0.18 (0.75)	-0.14 (1.06)
	CT	13	-0.26 (0.76)	0.53 (1.28)	-0.12 (0.88)
	TT	2	0.32 (0.54)	1.69 (0.08)	0.15 (0.83)
rs1800497 clinical risk	CC	26	0.05 (1.06)	0.03 (1.11)	0.17 (1.03)
	CT	11	0.03 (0.82)	0.38 (1.36)	0.32 (0.89)
	TT	1	0.49 (N/A)	0.25 (N/A)	1.76 (N/A)

## Discussion

We have noted associations between two DRD2 polymorphisms and cognitive function that differ depending on the presence of familial or clinical risk for psychotic disorders. Specifically, increasing numbers of the risk (C) allele at rs6277 was associated with better performance on a verbal cognitive factor, both in those with familial and clinical risk for psychosis, but not among population controls. The presence of one or more minor (T) allele at rs1800497 was associated with poorer performance on a psychomotor factor and this association interacted with the presence of familial risk, meaning it was only significantly associated with the psychomotor factor in those with familial risk for psychosis.

The rs6277 C allele at DRD2 was associated with better performance on a verbal factor in the present sample but subgroup analyses tentatively suggest that this association was only present in those with familial and clinical risk and was not present among population controls. Previous studies in healthy population samples have found that the C allele is associated with poorer performance on the Wisconsin Card Sorting Test [55] and working memory [56]. The C allele is associated with worse learning in young adults and worse overall cognitive ability in older adults. Broadly consistent with these findings, the results in our healthy control group suggested no association between the C or T allele and our verbal performance factor. It is perhaps surprising that we have found that, among those at risk for psychosis, the C allele associated with better performance on a “verbal” factor that includes vocabulary, verbal fluency and digit span testing. In addition to the cognitive associations mentioned above, the CC genotype has been associated with decreased striatal binding of DRD2 [29]. The significance of the C-allele may differ in clinical samples, including our psychosis risk groups, possibly in interaction with other factors. For example, homozygotes for the C allele have shown increased reward responsiveness after stress induction [57]. Differences in stress response between those at risk for psychosis and others may partly explain differences in verbal performance between groups.

The presence of one or more copies of the rs1800497 T allele was associated with poorer performance on a psychomotor factor in our sample but this association was only statistically significant in the familial risk group on subgroup and interaction analyses. The rs1800497 T allele has been associated with poorer cognitive performance across a range of domains. In addition, the T allele has been associated with a preference for slower motor speed [58] and slower reaction time in visual working memory [30]. We only found the association with our psychomotor factor (which loaded for the pegboard tasks primarily) in the family risk group. Poorer performance on this task only in those with familial risk may suggest increased genetic risk, possibly through interaction with another genetic factor. One possible candidate is the COMT-Val158Met polymorphism. Berryhill et al found the suggestion of differences in the association between rs1800497 and visual working memory according to COMT status [30].

Previous work in our sample, perhaps surprisingly, indicated that the risk groups did not differ in their neurocognitive performance from controls [41]. The authors suggested group similarities could be explained by the non-help-seeking nature of the risk samples and the recruitment of controls from the general population rather than a healthy sample. The present results suggest that there may be subgroups within the risk groups with intermediate phenotypes involving lower and higher neurocognitive performance. This would be consistent with findings in relation to the ZNF804A gene in a sample with schizophrenia [59]. Indeed, it is likely that the risk groups contain a variety of genetically-mediated intermediate phenotypes, two of which we have detected here. It remains to be determined if the group we have identified are at overall higher risk for psychosis based on their combined psychosis risk status and poorer cognitive performance.

We have identified risk variants for poorer cognitive outcomes that differ between those at risk for psychosis and population controls. These may be useful in predicting those at particularly high risk for more severe functional outcomes in a risk sample. If our observed associations are associated with longer-term illness consequences, this may help in highlighting a group at specific high risk within the familial and clinical risk groups. Furthermore, understanding how this risk interacts with genetic factors has the potential to inform prevention and treatment in the future, both in terms of clinical and neurocognitive outcomes.

This study has important strengths. First, the sample studied was recruited from a general population birth cohort study with considerable information available from Finnish national register data. The sample was therefore matched in terms of age, cultural background and was drawn from a relatively genetically homogeneous population in the north of Finland [60]. This limited the potential for confounding in the study. Furthermore, the control group who provided the reference ranges for neurocognitive scores reflected the broader population rather than a healthy volunteer group. Another interesting feature of our study is that our study group were a population risk group rather than a help-seeking risk group. This allows the study of a broader group with risk, including a large number of people who may not be studied in other situations. A further strength is that we were able to separately examine two risk groups, familial risk and clinical risk (prodromal syndrome). This study should also be interpreted in light of possible limitations. First, due to small sample size, some of these results, particularly results of subgroup analyses, may have arisen due to chance. The broader observed associations were relatively large, making chance an unlikely explanation for the findings. Furthermore, non-participation limited study numbers, which was important in limiting the power of the study. A further limitation is that, in the case of the rs1800497 SNP, the minor allele frequency was quite low (17%), resulting in low group numbers homozygous for the minor allele (5 in total). This was partly addressed by analysing the number of minor alleles as a continuous rather than categorical variable. Furthermore, the relatively genetically homogeneous nature of the population, while limiting generalizability of the results to other populations, limits the potential for confounding in the study. Nonetheless, it is important to recognise that results of subgroup analysis could have arisen due to chance.

In conclusion, we have identified markers that may increase risk for adverse cognitive outcomes in a community sample at risk (either familial or clinical) for psychotic disorders. Accordingly, we did not find these associations in a control group not at risk. Specifically, SNPs at the DRD2 gene may indicate risk for psychomotor factor performance in those with familial risk for psychosis and for verbal factor performance in those at familial and clinical risk for psychosis. They may therefore represent important identifiable intermediate phenotypes in those at risk.
